# Herbal medicine Guan Chang Fu Fang enhances 5-fluorouracil cytotoxicity and affects drug-associated genes in human colorectal carcinoma cells

**DOI:** 10.3892/ol.2014.2766

**Published:** 2014-12-03

**Authors:** CHEN YU, SHEN-LIN LIU, MING-HAO QI, XI ZOU, JIAN WU, JING ZHANG

**Affiliations:** 1 Department of Integrated Chinese and Western Medicine, The Affiliated Jiangsu Cancer Hospital of Nanjing Medical University and Jiangsu Institute of Cancer Research, Nanjing, Jiangsu 210009, P.R. China; 2 Senior Expert Consultation Center, Affiliated Hospital of Nanjing University of Chinese Medicine, Nanjing, Jiangsu 210029, P.R. China; 3 Department of Oncology, Affiliated Hospital of Nanjing University of Chinese Medicine, Nanjing, Jiangsu 210029, P.R. China; 4 Experimental Center, Affiliated Hospital of Nanjing University of Chinese Medicine, Nanjing, Jiangsu 210029, P.R. China; 5 Department of Electrocardiology, The Second Affiliated Hospital of Southeast University, Nanjing, Jiangsu 210003, P.R. China

**Keywords:** Guan Chang Fu Fang, colon cancer cell, apoptosis, synergy

## Abstract

Guan Chang Fu Fang (GCFF) is a natural compound, which is extracted from three medicinal plants, *Agrimonia pilosa* Ledeb*., Patrinia scabiosaefolia* and *Solanum nigrum* L*.* GCFF has demonstrated clinical efficacy in the treatment of colon cancer. At present, 5-fluorouracil (5-FU) is the primary active chemotherapeutic agent used for treating colon cancer. Using median-effect and apoptosis analyses, fluorescence microscopy and western blotting, the present study analyzed the association between GCFF and 5-FU in the human colon adenocarcinoma LoVo cell line. The effect of GCFF on the expression of chemotherapeutic agent-associated genes was also investigated. The results of the synergistic analysis revealed that GCFF exhibited a significant effect upon 5-FU-associated cytotoxicity within the LoVo cell line. This effect was observed over a broad dose-inhibition range (5–95%), but was particularly significant in the lower concentrations. The flow cytometry results revealed that low doses of GCFF or 5-FU induced S-phase arrest, as did a low-dose combination of the two drugs. After 48 h, GCFF significantly suppressed the expression levels of the chemotherapeutic agent resistance-associated genes within the colon cancer cells. The western blot analysis revealed that the combined effects of 5-FU and GCFF were due to a regulation of the B-cell lymphoma-2 family of proteins. The findings of the present study suggested that GCFF, when combined with 5-FU, has the potential to be a novel, chemotherapeutic compound for the treatment of colon cancer.

## Introduction

Colorectal carcinoma is one of the most prevalent forms of cancer that exists within Western countries (1), and is the second and third most common type of cancer in males and females, respectively. The majority of patients with advanced colon cancer require cytotoxic chemotherapy as a primary treatment (2). Recently, 5-fluorouracil (5-FU) has been widely used to treat cases of colon cancer. In addition, a number of attempts have been made to improve the objective response rates to chemotherapy, including the use of 5-FU in combination with other agents. However, the optimal combination regimen has not yet been identified, and the standard treatment modality remains debatable (3). Therefore, a requirement exists to identify novel compounds and optimized combined therapies for the treatment of colon cancer. A growing number of patients have selected herbal medicinal compounds as complementary therapies, in combination with conventional chemotherapeutic treatments (4). Due to the narrow therapeutic windows of existing chemotherapeutic drugs, these synergistic or additive interactions may improve the therapeutic results and decrease the necessary doses of current chemotherapeutic agents.

The Chinese herbal formula, Guan Chang Fu Fang (GCFF), contains ingredients from three medicinal plants, *Agrimonia pilosa* Ledeb., *Patrinia scabiosaefolia* and *Solanum nigrum* L. serve as adjuvants to assist the effects of the primary ingredient, *A. pilosa*. In traditional Chinese medicine, *A. pilosa* is a plant that possesses anti-cancer (5), anti-oxidant (6), acetylcholinesterase inhibitory (7) and anti-inflammatory (8) activities. Certain studies have identified that *A. pilosa* contains the phenolic compounds catechin, agrimonin and quercetin (9). However, ethanol extracts of *A. pilosa* have not yet been examined. *P. scabiosaefolia*, another component of GCFF, has also been used in Chinese medicinal formulas for the treatment of carbuncles, stasis, intestinal abscess and dysmenorrhea (10). Furthermore, *P. scabiosaefolia* is also an important component of formulated traditional Chinese medicine prescriptions to treat gastrointestinal and breast cancer (11). In addition, a number of *in vitro* studies have revealed that *Solanum nigrum* L. has antitumor effects against various types of cancer, including leukemia and stomach, colon and endometrial cancers (12). Further studies indicated that an aqueous extract of *Solanum nigrum* L. was able to enhance the cytotoxicity of 5-FU, docetaxel, cisplatin and doxorubicin in colorectal cells (13). Due to the variety of adjuvant components, each herbal formula has a different name. The term Guan Chang Fu Fang, meaning ‘enema of compound’ in Chinese, was derived from the fact that the compound is clinically used for enemas. Our preliminary experiments confirmed that the ethanol extract of GCFF was more effective than the aqueous extract (14), which led to the use of the ethanol extract within the present study. *In vitro* and *in vivo* studies have revealed that each component of the GCFF compound has a significant cytotoxic effect upon numerous types of cancer, particularly cancers of the digestive system (12). Despite this, the role of GCFF in the treatment of cancer has not yet been addressed by modern science. Therefore, the present preclinical study aimed to investigate whether the combination of GCFF and 5-FU could produce a significant synergistic interaction, which could treat colon cancer. Furthermore, the expression of chemotherapeutic agent resistance-related genes in colon cancer cells following treatment with GCFF and 5-FU, either alone or in combination, was investigated.

## Materials and methods

### Preparation of the GCFF extract

The medicinal plants used for the preparation of the GCFF extract were provided by Bozhou Yonggang Medicinal Herbs Factory Co., Ltd., (Bozhou, China). The preparation included obtaining the ethanol extracts from the crude plant ingredients of *A. pilosa*, *P. scabiosaefolia* and *Solanum nigrum* L., at a ratio of 5:1:1. The plants were homogenized with a Waring blender (Shanghai Specimen Model Factory, Shanghai, China), and then soaked at a 10:l dilution in double-distilled water for 24 h. The mixture was then heated to 100°C for 2 h, after which an 8-fold volume of distilled water was added, followed by further heating for 1.5 h. Next, the residue from the two combined extracts was extracted twice with 80% ethanol. Firstly, the plant-extract residue was extracted in a 10-fold volume of ethanol for 2 h, and then an 8-fold volume of 80% ethanol was added. The mixture was heated for a further 1.5 h, prior to the merging of the two extracts, and then heated to 70°C to evaporate the ethanol. Next, the ethanol extract was concentrated, and the decoction was filtrated. For GCFF, the raw ethanol extract was mixed at a concentration of 1.4 g herb/ml, and then filtered through a 0.2-mm filter (Microgen, Laguna Hills, CA, USA) prior to use. The quality control of the GCFF preparation, including definition of the correct plants, production origin, implantation, harvesting and processing, was conducted according to the guidelines defined by Nanjing Herb Pharmaceutics, Ltd. The species, plant parts and origins used within the GCFF formula are revealed in Table I. The total weight of the boiled herbs was 210 g.

### Cell lines and cell culture

The poorly-differentiated human colon adenocarcinoma LoVo cell line was provided by the Center Laboratory of the Jiangsu Province Chinese Hospital (Nanjing, China). The LoVo cell lines were propagated in RPMI-1640 medium (Gibco-BRL, Carlsbad, CA, USA), which was supplemented with 10% bovine serum, 100 U/ml penicillin and 100 μg/ml streptomycinat 37°C in a water-saturated atmosphere with 5% CO_2_.

### Drugs

5-FU was supplied by the Jiangsu Hengrui Medicine Company (Jiangsu, China). The Cell Titer 96 AQueous One Solution Cell Proliferation Assay kit was purchased from Promega (Madison, WI, USA) and the Annexin V-fluorescein isothiocyanate (FITC) Apoptosis Detection kit was purchased from Invitrogen (Carlsbad, CA, USA).

### Cytotoxicity assay and analysis of combination effects

The LoVo tumor cells were grown until the log-phase had been reached, and then were seeded at a density of 8×10^3^ cells per well into 96-well plates. The RPMI-1640 medium in each well was replaced with fresh medium, or with medium containing various drug concentrations (0.21, 0.43, 0.87, 1.75, 3.5 and 7 mg/ml GCFF, and 0.02, 0.04, 0.16, 0.64, 2.5 and 10 μg/ml 5-Fu, as a single drug or in combination), for 48 h. The cells were incubated for an additional 4 h with MTT, prior to absorbance analysis at 490 nm using a microplate reader (elx800; Bio-Tek Instruments, Inc., Winooski, VT, USA). The cell growth inhibition rate was calculated using the following formula: Inhibition rate = 1 − OD_experiment_/OD_control_, where OD is the optical density. The dose-response curves were obtained for GCFF: 0.21, 0.43, 0.88, 1.75, 3.5 and 7 mg/ml and 5-FU: 0.02, 0.04, 0.16, 0.64, 2.5 and 10 μg.ml, alone, and for multiple dilutions of fixed-ratio combinations of the two drugs (0.02:0.21, 0.04:0.43, 0.16:0.88, 0.64:1.75, 2.5:3.5 and 10 μg/ml:7 mg/ml, 5-FU:GCFF, respectively). The median-effect analysis was performed using the combination index (CI) method, according to Chou and Talalay (15). The CI is defined by the following equation: CI = (D)_1_/(Dx)_1_ + (D)_2_/(Dx)_2_ + α(D)_1_(D)_2_/(Dx)_1_(Dx)_2_. (Dx)_1_ and (Dx)_2_ are the concentrations of D_1_ (GCFF) and D_2_ (5-FU) alone, which give x% inhibition, whereas (D)_1_ and (D)_2_, as the numerators, are the concentrations of GCFF and 5-FU that produce an identical effect level when in combination. For example, α=0 when GCFF and 5-FU are mutually exclusive (with similar modes of action), whereas α=1 when GCFF and 5-FU are mutually non-exclusive (with independent modes of action). A CI level of >1 indicates antagonism, whereas a CI level of <1 indicates synergy and a CI level equal to 1 indicates additivity. The CI ratio represented in the present study was the mean value derived from at least three independent experiments.

### Apoptosis assay

The LoVo cells were briefly plated on a 60-mm Petri dish and allowed to grow to reach 75–80% confluence. The cells were then exposed to GCFF and 5-FU, either alone or in combination, for 48 h. Following incubation, the tumor cells were compared with the untreated control cells. Next, the cells were collected and resuspended in 500 μl binding buffer, to which 5 μl each of Annexin V-FITC and propidium iodide (PI) was added. The analyses were performed on a flow cytometer (FACScalibur; BD Biosciences, Franklin Lakes, NJ, USA).

### Cell cycle analysis

In total, 1×10^5^ cells were seeded into 6-well plates and incubated overnight. The cells were then treated with with 0.43 mg/ml GCFF and 0.04 μg/ml 5-FU, as a single drug or in combination), for 48 h. Next, the cells were harvested, washed with cold phosphate-buffered saline (PBS) and then fixed for 12 h with 70% ethanol in PBS at 4°C. Following incubation, the cells were resuspended in PBS with 100 μg/ml RNase and 50 μg/ml PI, and incubated at 37°C for 30 min. The cell cycle distribution of nuclear DNA was determined by flow cytometry using an FC500 cytometer (Beckman Coulter Inc., Pasadena, CA, USA).

### Fluorescence microscopy

In total, 1×10^6^ LoVo cells were seeded into 6-well plates, incubated overnight and then treated with either 0.43 mg/ml GCFF, 0.04 μg/ml 5-FU or a combination of GCFF and 5-FU for 48 h. The cells were then washed twice with PBS, fixed overnight with cold methanol and acetic acid at a ratio of 3:1, and then stained with 1 μg/ml Hoechst 33342 (Life Technologies, Carlsbad, CA, USA) for 30 min in the dark. The stained cells were observed using a fluorescence microscope (magnification, ×400; IX51; Olympus Corporation, Tokyo, Japan).

### Reverse transcription quantitative polymerase chain reaction (RT-qPCR)

The total RNA was isolated using TRIzol reagent (Life Technologies), and reverse-transcribed into cDNA using a RT reagent kit (Takara Bio, Inc., Shiga, Japan). The PCR reactions were performed using the ABI 7500 fast real-time PCR system (Life Technologies) and 1X ABsolute QPCR Mix (Thermo Fisher Scientific, Waltham, MA, USA). The sequences of the primers (GenScript USA Inc., Piscataway, NJ, USA) were as follows: Orotate phosphoribosyl transferase (OPRT) forward, 5′-CGAGTAAGCATGAAA CCAGA-3′ and reverse, 5′-CTACTCAAATACGCTTCC CCA-3; thymidylate synthase (TS) forward, 5′-ACCTGAATC ACAATCGAGCCA-3′ and reverse, 5′-TTGGATGCGGATTGT ACCCT-3′; dihydropyrimidine dehydrogenase (DPD) forward, 5′-TGTTCGGACAGAGCAAGATG-3′ and reverse, 5′-CTF CAATCCGGCCATITCTA-3′; and glyceraldehyde 3-phosphate dehydrogenase (GAPDH) forward, 5′-CCATGGAGA AGGCTGGGG-3′ and reverse, 5′-CAAAGTTGTCATGGA TGACC-3′. The PCR conditions were 50°C for 2 min and 95°C for 15 min, followed by 45 cycles at 95°C for 15 sec and 60°C for 1 min. The relative gene expression quantifications were calculated according to the comparative CT method, using glyceraldehyde 3-phosphate dehydrogenase as an endogenous control and commercial human total RNA (Clontech Laboratories Inc., Mountain View, CA, USA) as a calibrator. The final results were determined according to the 2^−ΔΔCT^ method (16).

### Western blot analysis

In total, 1×10^6^ LoVo cells were seeded into 6-well plates, and incubated overnight. The cells were treated according to the aforementioned instructions. Next, the cells were washed twice with ice-cold PBS. The total proteins were solubilized and extracted using a lysis buffer, which consisted of 20 mM HEPES (pH 7.9), 20% glycerol, 200 mM KCl, 0.5 mM EDTA, 0.5% NP40, 0.5 mM DTT and 1% protease inhibitor cocktail. The protein concentrations were determined using a bicinchoninic acid protein assay. The samples were separated using SDS-PAGE, and then transferred to polyvinylidene fluoride membranes by electroblotting, prior to antibody probing with rabbit anti-human B-cell lymphoma-2 (Bcl-2)-associated X protein (Bax) polyclonal antibody (1:1,000; Cell Signaling Technology, Inc., Danvers, MA, USA), rabbit anti-human Bcl-2 polyclonal antibody (1:1,000; Santa Cruz Biotechnology, Inc., Santa Cruz, CA, USA) and rabbit anti-human Bcl-2 19-kDa interacting protein 3 (Bnip3) polyclonal antibody (1:1,000; Santa Cruz). The membranes were then incubated with goat anti-rabbit IgG-horseradish peroxidase secondary antibodies (dilution, 1:10,000; Cell Signaling Technology, Inc.). The blots were developed with an enhanced chemiluminescence kit, and each western blot assay was repeated three times.

### Statistical analysis

The values are expressed as the mean ± standard deviation. The statistical comparisons were performed using Student’s t-test. P<0.05 was used to indicate a statistically significant difference.

## Results

### Cytotoxicities of GCFF and 5-FU against LoVo cells

The cytotoxic activities of GCFF and 5-FU were investigated individually. As expected, GCFF and 5-FU individually inhibited the proliferation of the LoVo cells in a dose-dependent manner. Table II reveals the half maximal inhibitory concentration (IC_50_) doses for the LoVo cells upon exposure to GCFF or 5-FU. The response of the LoVo cells to the different drugs were significantly different (P=0.003).

### Median-effect analysis of combined GCFF and 5-FU in vitro

**Figure 1:**
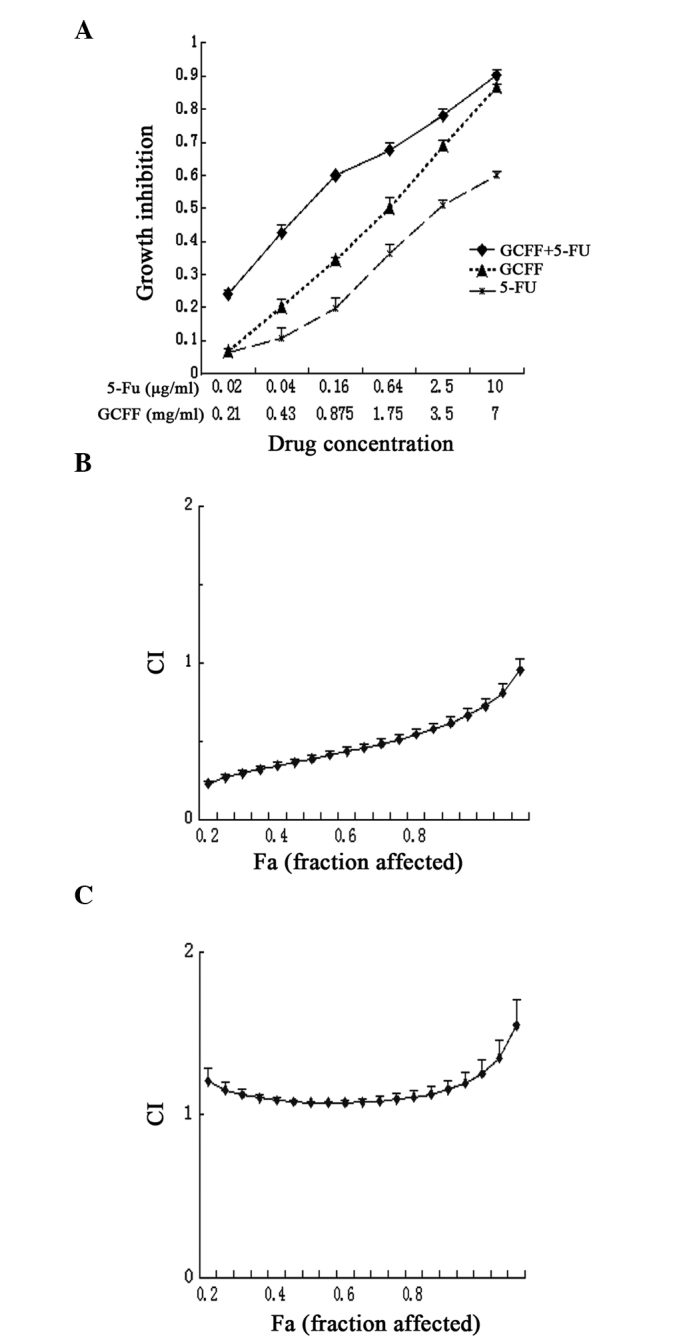
(A) Dose-response curves of the human colon adenocarcinoma LoVo cells treated with Guan Chang Fu Fang (GCFF) and 5-fluorouracil (5-FU), alone or in combination. (B) Combination index (CI) values of the LoVo cells at different levels of growth inhibition (as represented by the fraction affected) induced by GCFF plus 5-FU. (C) CI values according to the treatment schedule of LoVo cells with the chemotherapeutic agents, with administration of 5-FU preceding that of GCFF. The cells were pretreated with 5-FU for 24 h, followed by the administration of GCFF at fixed ratios for 48 h. The data points indicate the means of at least three independent experiments, and the bars indicate the standard deviations. CI<1, =1 and >1 indicate synergism, addition and antagonism, respectively.

Fig. 1A presents the dose-response curves for the LoVo cells that were exposed to GCFF and 5-FU, alone or in combination. The combination of GCFF and 5-FU demonstrated significant proliferative inhibition of the LoVo cells at the majority of doses (0.21–3.5 mg/ml GCFF) (P=0.008). Fig. 1B reveals the cytotoxic effect upon the cells simultaneously treated with GCFF and 5-FU. As the CI values were below a relatively broad range of killed cell fractions, this suggested that GCFF exhibited a synergistic effect upon the cytotoxicity of 5-FU over a broad dose-inhibition range. In addition, the present study analyzed the effect of sequential drug delivery upon the LoVo cells; GCFF or 5-FU were administered alone for 24 h, prior to administration of the second drug. The treatment schedule in which GCFF was administered prior to 5-FU demonstrated a synergistic growth inhibitory effect, similar to that observed in the simultaneous treatment regimen. However, significant antagonistic effects were identified when the cells were treated in the reverse order (P=0.01; Fig. 1C). These results indicated that the simultaneous treatment and administration of GCFF prior to 5-FU was more effective compared with the reverse order.

### Apoptotic effects mediated by GCFF and 5-FU

**Figure 2:**
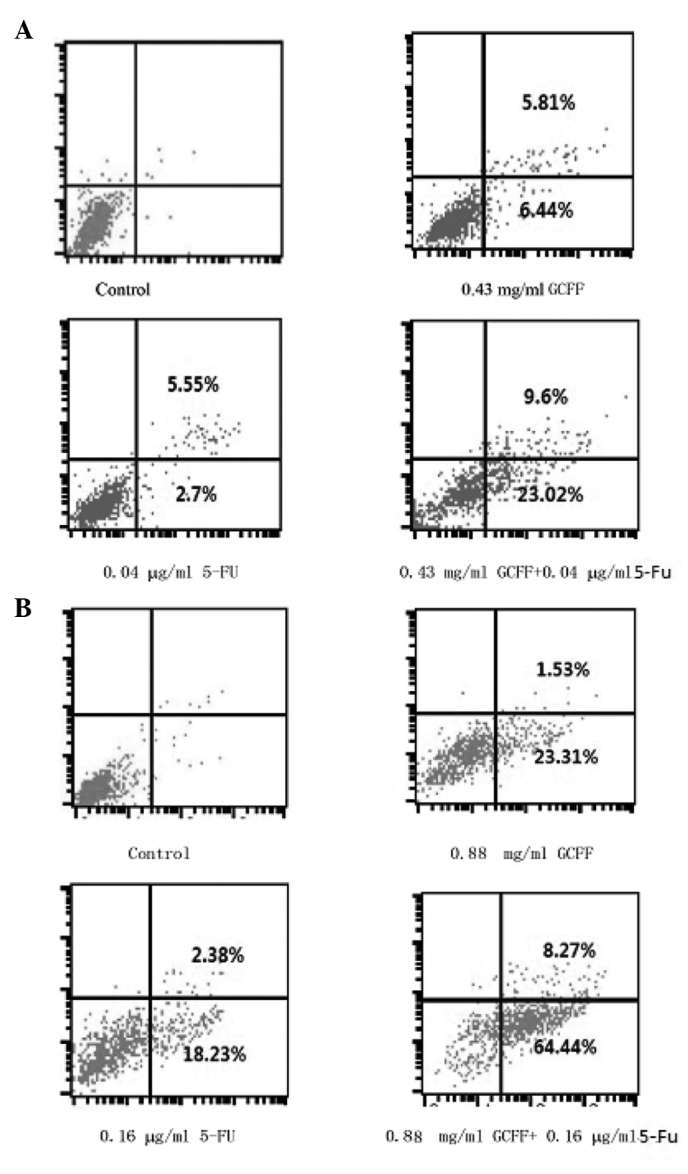
Annexin V-fluorescein isothiocyanate and propidium iodide double-staining to analyze the apoptotic status of the treated human colon adenocarcinoma LoVo cell line. (A) Low-dose drug and (B) moderate-dose drug. GCFF, Guan Chang Fu Fang; 5-FU, 5-fluorouracil.

The Annexin V-FITC and PI double-staining was performed in order to distinguish between the apoptotic cells and the other cell populations. The LoVo cell lines were treated with GCFF and 5-FU, alone and in combination. The percentage of apoptotic cells present following treatment with 5-FU was significantly increased by the co-administration of GCFF (P=0.003). This indicated that the simultaneous treatment of GCFF and 5-FU induced apoptosis in a synergistic manner (Fig. 2). In particular, the percentages of LoVo cells that had undergone early apoptosis, induced by single-agent treatment with either GCFF and 5-FU, were 23.31 and 18.23%, respectively, whereas the percentage of apoptotic cells following the combined drug regimen increased to 64.44%.

### GCFF induces S-phase cell cycle arrest in LoVo cells

**Figure 3:**
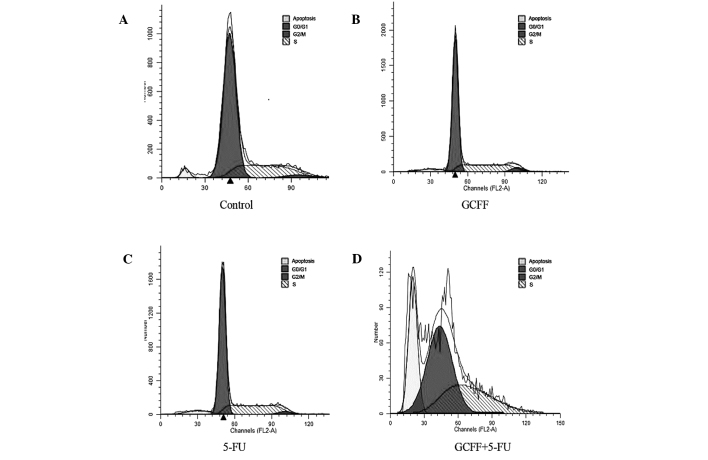
Cell cycle arrest in human colon adenocarcinoma LoVo cells following 48 h of treatment. (A) Untreated control; (B) Guan Chang Fu Fang (GCFF); (C) 5-fluoroucil (5-FU) and (D) GCFF+5-FU.

Due to the significant effect of the GCFF and 5-FU co-administration upon the apoptotic rate of the LoVo cells, the present study also examined the potential effects of the combined GCFF and 5-FU regimen upon the cell cycle distribution of LoVo cells at doses below IC_30_. As revealed in Fig. 3 and Table III, the LoVo cells treated with 0.43 mg/ml GCFF and 0.04 μg/ml 5-FU demonstrated a larger number of cells in the S-phase (38.06±1.90%) compared with the cells treated with GCFF alone (27.99±0.38%). Furthermore, at lower doses, treatment with GCFF increased the number of cells in the S-phase of the cell cycle. Therefore, the results of the present study suggested that following a 48-h treatment with a low-dose combination of the two drugs, the cells were arrested in the S-phase of the cell cycle.

### Percentage of apoptotic cells induced by combination therapy is significantly higher compared with monotherapy

**Figure 4:**
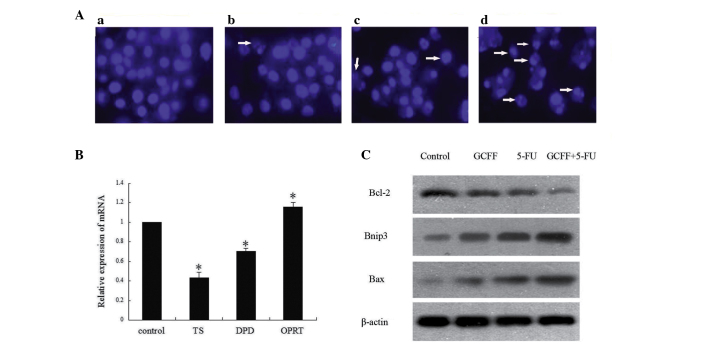
(A) Hoechst 33342 staining revealing the presence of apoptotic cells. The human colon adenocarcinoma LoVo cells were (a) left untreated or treated for 48 h with either (b) 0.43 mg/ml Guan Chang Fu Fang (GCFF), (c) 0.04 μg/ml 5-fluoroucil (5-FU) or (d) GCFF + 5-FU. Compared with the control group, the GCFF- and 5-FU-treated cells exhibited chromatin condensation, nuclear fragmentation and apoptotic bodies (white arrows). (B) GCFF suppressed the mRNA expression of certain chemotherapeutic agent resistance-related genes (TS, DPD and OPRT) in the LoVo cells. The changes in the relative gene expression levels following a 48-h treatment with the half maximal inhibitory concentration of GCFF are revealed (^*^P<0.05 vs. the control). (C) Western blot analysis revealing the combined effects of GCFF and 5-FU upon the expression of members of the B-cell lymphona-2 (Bcl-2) family of proteins. LoVo cells were treated with 0.43 mg/ml GCFF, 0.04 μg/ml 5-FU or GCFF + 5-FU for 48 h. β-actin served as the control. Bnip3, Bcl-2 19-kDa interacting protein 3; Bax, Bcl-2-associated X protein; TS, thymidylate synthase; DPD, dihydropyrimidine dehydrogenase; OPRT, orotate phosphoribosyl transferase.

Following a 48-h incubation with either 0.43 mg/ml GCFF, 0.04 μg/ml 5-FU or a combination of the two, the cells were examined by fluorescence microscopy. The chromatin condensation, nuclear fragmentation and apoptotic bodies were clearly identified in the treated cells (Fig. 4A). Compared with the monotherapy-treated cells, the percentage of apoptotic cells increased significantly following treatment with the combined therapy (P=0.005).

### GCFF affects the mRNA expression of chemotherapeutic agent resistance-related genes

To explain the mechanisms that underlie the synergistic association between GCFF and 5-FU, the present study hypothesized that GCFF may affect the expression of certain chemotherapeutic agent resistance-related genes, namely OPRT, TS and DPD, which could effect the sensitivity of the LoVo cells to 5-FU. The cells were incubated for 48 h with GCFF or 5-FU alone, at their respective IC_40_ values, or in combination. As revealed in Fig. 4B, the expression levels of TS and DPD were significantly downregulated following treatment with GCFF alone. By contrast, the expression levels of OPRT were significantly upregulated by GCFF. However, administration of 5-FU alone did not downregulate the expression of the drug resistance-associated genes.

### Combined effect of GCFF and 5-FU on the expression of the Bcl-2 family of proteins

In order to investigate the molecular mechanisms that underlie the combined anticancer effects of GCFF and 5-FU, the effects of GCFF and 5-FU, alone or in combination, upon the expression of the Bax, Bcl-2 and Bnip3 were investigated. As shown in Fig. 4C, the western blot analysis demonstrated that treatment with either GCFF or 5-FU alone reduced Bcl-2 expression, and increased Bax and Bnip3 expression. Furthermore, the combined GCFF and 5-FU treatment significantly reduced Bcl-2 expression, and increased Bax and Bnip3 expression.

## Discussion

The use of active combination chemotherapy has the potential to reduce drug toxicity and dosages, and address the issue of drug resistance. The aim of the present study was to investigate whether the cytotoxic effect of 5-FU could be enhanced by the Chinese herbal medicinal compound, GCFF; this was determined using media-effect analysis, flow cytometry and fluorescence microscopy. The results of the present study indicated that the simultaneous administration of GCFF and 5-FU inhibited cell growth and induced apoptosis in a synergistic manner within the LoVo cell line. It was concluded that GCFF contributed to 5-FU-induced apoptosis and growth inhibition. Next, low doses of GCFF and 5-FU were revealed to induce S-phase cell cycle arrest within the LoVo cells via the Bcl-2 family of proteins. Finally, the expression levels of certain chemotherapeutic agent resistance-related genes were identified to be downregulated by GCFF alone and in combination with 5-FU. Overall, the results indicated that GCFF may be a potential candidate for a combined therapy approach alongside 5-FU. The underlying therapeutic mechanisms of this combined therapy may be attributable to a downregulation of certain chemotherapeutic agent resistance-related genes and a synergistic effect upon the rate of cellular apoptosis. Complementary and alternative medicines have been increasingly accepted by patients with cancer in China (6), and a number of patients have taken herbal medicines prior to or during chemotherapy. One previous study revealed that certain herbal medicinal formulae improve the clinical outcomes of chemotherapy (17). However, the potential underlying mechanisms have not yet been identified. Another prior study demonstrated the potential of a number of plant-derived compounds to sensitize tumor cells to chemotherapeutic agents and restore the sensitivity of certain drug-resistant cells (16). However, further elucidation was required as to whether the herbal medicinal formulae could sensitize tumor cells to chemotherapeutic agents. In the present study, a media-effect analysis was performed in order to observe the interaction between GCFF and 5-FU in the LoVo cell lines. In the future, similar studies could be performed upon other cell lines to further investigate the therapeutic potential of combination regimens against different types of tumor cells.

In recent years, 5-FU has been widely administered for the treatment of colon cancer (18). 5-FU is able to induce DNA damage, either directly or indirectly (19), and initiate apoptosis via the p53-dependent pathway (20). A number of experimental studies have revealed that the overexpression of chemotherapy agent resistance-related genes is associated with drug resistance. The DNA synthase enzyme, TS, is targeted by 5-FU and possesses an important role in the efficacy of 5-FU. High levels of TS expression have been demonstrated to contribute to the occurrence of 5-FU resistance and poor clinical outcomes (21). Certain *in vivo* and *in vitro* studies have revealed that OPRT is one of the key enzymes involved in 5-FU metabolism, and that phosphorylation of its active metabolite is necessary to inhibit cellular DNA synthesis and induce RNA dysfunction (22). In addition, OPRT mRNA expression has been demonstrated to predict 5-FU sensitivity; specifically, patients with high OPRT expression are more sensitive to 5-FU treatment, and are therefore more likely to benefit from it (23). DPD is an enzyme that catalyzes the major catabolic step during pyrimidine metabolism (24). A previous study identified that low levels of DPD were associated with low TS expression, and were also correlated with responses to 5-FU-based chemotherapy (25). Further studies have revealed that, in addition to being an important determinant of 5-FU pharmacokinetics and clinical toxicity, DPD activity is also a significant factor involved in the determination of 5-FU availability for the production of active metabolites within tumors (26). This finding highlights the potential of DPD activity to predict the response of tumors to 5-FU therapy. In the present study, TS and DPD mRNA were revealed to be significantly downregulated by GCFF, alone or in combination with 5-FU. By contrast, treatment with 5-FU alone did not downregulate the expression of the resistance-associated genes. This finding may explain why GCFF was able to sensitize the cells to 5-FU, and why the synergistic effect of the simultaneous treatment regimen and the administration of GCFF prior to 5-FU, differed from the effect observed upon the administration of 5-FU prior to GCFF.

The Bcl-2 family of proteins are key regulators of the apoptotic pathway (19). The results of the present study revealed that treatment of the LoVo cells with GCFF, in combination with 5-FU, significantly decreased the expression of Bcl-2, but increased the expression of Bax and Bnip3. This finding indicated that GCFF and 5-FU induce apoptosis by regulating the expression of the Bcl-2 family of proteins. The mitochondrial protein Bnip3, formerly known as NIP3, is a member of the Bcl-2 family that induces apoptosis via the mitochondrial permeability transition pore in the absence of a functional BH3 domain. In normal tissues, endogenous Bnip3 is loosely associated with the mitochondrial membranes, but during the induction of apoptosis, it is fully integrated within the outer mitochondrial membrane, with the N-terminus remaining in the cytoplasm and the C-terminus in the membrane (27). The Bcl-2 proteins, in particular Bnip3, mediate the balance between pro- and anti-apoptotic actions. This balance has a significant role in tumor evolution processes. Further analysis, through the examination of gene expression profiles of these different pathways, is required.

## Figures and Tables

**Table I:** Guan Chang Fu Fang components.

Family	Latin binomial	Plant part	Origin
Rosaceae	*Agrimonia pilosa* Ledeb.	Everything above ground	Hubei, China
Valerianaceae	*Patrinia scabiosaefolia*	Root	Sichuan, China
Solanaceae	*Solanum nigrum* L.	Everything above ground	Anhui, China

**Table II:** IC_50_ doses of GCFF and 5-FU.

	IC_50_ (mean ± SD)	
	
Cell line	GCFF, mg/ml	5-FU, μg/ml
LoVo	1.62±0.09	2.91±0.46

IC_50_, half maximal inhibitory concentration; GCFF, Guan Chang Fu Fang; 5-FU, 5-fluorouracil; SD, standard deviation.

**Table III:** Percentage of cells in each phase of the cell cycle, and total percentage of apoptotic cells, following 48 h of incubation.

Drug (concentration)	Cell cycle phase (% of cells)	% of apoptotic cells
Control	G_0_/G_1_ (71.45)	
	S (25.76)	
	G_2_/M (2.79)	
GCFF (0.43 mg/ml)	G_0_/G_1_ (67.81)	5.08
	S (27.99)	
	G_2_ (4.25)	
5-FU (0.04 μg/ml)	G_0_/G_1_ (67.33)	5.47
	S (30.08)	
	G_2_/M (2.59)	
GCFF (0.43 mg/ml) + 5-FU (0.04 μg/ml)	G_0_/G_1_ (60.98)	
	S (38.06)	24.92
	G_2_/M (0.97)	

GCFF, Guan Chang Fu Fang; 5-FU, 5-fluorouracil.

## Abbreviations

GCFF: Guan Chang Fu Fang
5-FU: 5-fluorouracil
OPRT: orotate phosphoribosyl transferase
TS: thymidylate synthase
DPD: dihydropyrimidine dehydrogenase gene
CI: combination index
PI: propidium iodide
